# Plasma Levels of Matrix Metalloproteinases 2 and 9 in Patients with Chronic Chagas Heart Disease and Systemic Arterial Hypertension: Correlation with TGF-Beta Plasma Levels

**DOI:** 10.1155/2023/8484697

**Published:** 2023-04-19

**Authors:** Reinaldo B. Bestetti, Renata Dellalibera-Joviliano, Ellen Rizzi, Giselle F. Bonacio, Milton Faria-Jr, Rosemeire Furlan-Daniel, Suzeley Castro-França

**Affiliations:** ^1^Department of Medicine, UNAERP Medical School, University of Ribeirão Preto, Ribeirão Preto, Brazil; ^2^Department of Biotechnology, University of Ribeirão Preto, Ribeirão Preto, Brazil

## Abstract

**Background:**

Chronic Chagas heart disease (CCHD) and systemic arterial hypertension (SAH) frequently coexists in areas where Chagas disease is endemic. The effects of the association of both conditions (CCHD-SAH) on the extracellular matrix (ECM) remodeling are unknown. Matrix metalloproteinases (MMP) 2 and 9 are involved in ECM remodeling. The aim of this study was to evaluate MMP 2 and MMP9 in CCHD-SAH patients and to correlate their levels with those of the profibrogenic cytokine TGF-beta.

**Methods:**

We included 19 patients with CCHD-SAH, 14 patients with CCHD alone, and 19 controls matched by sex and age. MMP-2 and MMP-9 plasma levels were studied by gel zymography and showed as optical densities (OD). TGF-beta plasma levels were measured by double-ligand ELISA and expressed as pg/mL.

**Results:**

Median (5^th^, 95^th^) MMP-2 plasma levels were 1224.7 OD (1160, 1433.5) in patients with CCHD alone, 1424.1 OD (1267.5, 1561) in patients with CCHD-SAH, and 940 OD (898.1, 1000.8) in controls (*p*=0.001). MMP-9 plasma levels were 1870 OD (1740, 1904.1) in patients with CCHD alone, 1754.6 OD (1650, 2049) in those with CCHD-SAH and 89.7 OD (80, 96) in controls (*p*=0.0003). MMP-9 plasma levels were higher than those of MMP 2 in patients with CCHD-SAH (*p*=0.01). No correlation was found between TGF-beta plasma levels with MMP-2 serum levels (*r* = 0.12; *p*=0.7), but a moderate negative correlation (*r* = −0.46; *p*=0.048) was observed between TGF-beta and MMP-9 plasma levels.

**Conclusions:**

MMP-2 and especially MMP-9 may play a role in the ECM remodeling process in patients with CCHD-SAH. TGF-Beta may counteract the MMP effect on the ECM remodeling process in patients with CCHD-SAH.

## 1. Introduction

Chagas disease is a major health problem in Latin America, where it affects about 6 million people, and other 100 million people are at risk of acquiring the disease [1]. Nowadays, Chagas disease is an emergent health problem in areas where the disease is nonendemic because of immigration [2]. The disease is caused by the protozoan *Trypanosoma cruzi*, which is transmitted to humans via the feces of a kissing bug (*Triatoma infestans*). Up to two decades after infection, about 20% of infected patients develop chronic cardiomyopathy.

Chagas cardiomyopathy is clinically characterized by malignant ventricular arrhythmias [3], precordial chest pain with acute myocardial infarction without obstructive coronary artery disease [4], thromboembolic phenomena, sudden cardiac death, heart failure with reduced left ventricular ejection fraction (HFREF), and bundle branch blocks and AV blocks [5]. Chagas cardiomyopathy with HFREF has the worst prognosis among other causes of mild to severe HFREF [6, 7]; at the terminal stages, heart transplantation is the treatment of choice [8].

In areas where chronic Chagas disease is endemic, systemic arterial hypertension (SAH) is frequently observed, affecting up to 33% of patients with chronic Chagas disease (CCD) [9] In turn, the presence of SAH may pose patients with CCD at risk to develop cardiomyopathy (CCHD-SAH); in fact, HFREF can be found in about 31% of patients with CCHD-SAH [10].

The pathogenesis of CCHD-SAH is poorly understood. Only two previous studies have addressed the question of myocardial inflammation in CCHD-SAH by measuring cytokine plasma levels; these studies have shown conflicting results [11, 12]. Proinflammatory cytokines may induce extracellular matrix (ECM) remodeling by partially interfering with matrix metalloproteinases (MMP) [13].

MMPs are a set of enzymes that play a pivotal role in the homeostasis of the ECM. They are responsible for the degradation of excessive molecules of the ECM (collagen IV, fibronectin, and laminin) synthetized under pathological conditions [14], as well as for the degradation of cardiomyocyte myosin heavy chain in cardiomyopathy [15]. Increased levels of soluble MMPs, such as MMP-2 and MMP-9, have been detected in the plasma of patients with several types of heart disease, including hypertensive heart disease [16] and ischemic heart disease [17]. Increased plasma levels of MMP-2 [18–20] and/or MMP-9 [13, 18] have been found in patients with HFREF. As far as we know, only one previous study concerning MMP plasma levels measurement has been carried out in the setting of CCHD-SAH [21].

Plasma levels of the anti-inflammatory cytokine TGF-beta have been found to be increased in patients with CCHD-SAH [11]. In non-Chagas disease patients, plasma levels of TGF-beta have an inhibitory effect on MMPs [22]. To our knowledge, the association between MMP and TGF-beta serum levels has not been investigated in patients with CCHD-SAH. Accordingly, the aim of this investigation was to measure MMP plasma levels, and correlated them with TGF-beta plasma serum levels, in patients with CCHD-SAH in an attempt to expand our understanding of the pathogenesis of this condition.

## 2. Methods

### 2.1. Patients

Patients routinely followed at the cardiomyopathy outpatient service were recruited for this work. This cross-sectional study enrolled 19 patients with CCHD-SAH (11 men and 8 women; mean age 62 ± 2 years), 14 patients with CCHD alone (9 men and 5 women; mean age 63 ± 2 years), and 19 normal subjects that served as controls. The characteristics of the controls were as follows: 10 men and 9 women; mean age 62 ± 2 years; all of them with a negative serology for Chagas disease; no chronic and/or infectious and/or inflammatory diseases; no continuous medication use; matched by age and sex.  Table 1 summarizes these findings. The diagnosis of chronic Chagas disease was made on the basis of two positive serologies (hemagglutination, ELISA, or immunofluorescence). The diagnosis of CCHD-SAH was made in patients with positive serology for chronic Chagas disease and SAH >140 mm HG or >90 mmHg. Patients with normal systemic arterial pressure upon admission, but taking antihypertensive medication and with a documented SAH was considered to have CCHD-SAH as well, as previously described [23,24]. Except for SAH, patients with any other disease that could induce the appearance of heart disease by itself were ruled out from the investigation. Patients with diabetes mellitus were also ruled out because of the potential impact of this disease on cytokine plasma levels.

The work-up consisted of history-taking, physical examination upon admission, 12-lead resting ECG, and 2-D echocardiography. Patients with ECG abnormalities and/or echocardiographic pathological changes and normal systemic arterial pressure were said to have CCHD; those with such abnormalities and SAH were diagnosed as having CCHD-SAH.

Patients with left ventricular systolic dysfunction (LVSD) were treated with angiotensin converting enzyme inhibitors (ACEI)/angiotensin receptor blocks (ARB), betablockers, and aldosterone inhibitor. Patients with CCHD-SAH without LVSD were treated at the discretion of the attending physician with antihypertensive medication, preferentially with ACEI/ARB drugs. Therefore, all patients with CCHD-SAH were treated with antihypertensive medication.

ACEI/ARB were given to 7 (50%) out of 14 patients with CCHD and to 17 (89%) out of 19 patients with CCHD-SAH (*p*=0.26). Betablockers were given to 4 (28%) out of 14 patients with CCHD, and to 7 (37%) out of 19 patients with CCHD-SAH (*p*=0.21). Spirolactone was used in 2 (14%) out of 14 patients with CCHD, and in 7 (37%) out of 19 patients with CCHD-SAH (*p*=0.23). Abnormalities in the 12-lead ECG were found in 13 (93%) patients with CCHD, and in 17 (89%) patients with CCHD-SAH (*p*=0.29). Reduced LVEF was observed in 13 (98%) of 14 CCHD patients, and in 16 (86%) of 19 patients with CCHD-SAH (*p*=0.10). The mean LVEF was 53.5 ± 1.3% in patients with CCHD and 54 ± 1.60% in patients with CCHD-SAH (*p*=0.71).

The study was approved by the Ethics Committee (CAAE 48541715.5.0000.5498) and cope with the declaration of Helsinki. After the written informed consent was obtained, the measurements were performed.

### 2.2. Cytokine (TGF-Beta) Essay

Blood was collected from the antecubital vein, the plasma separated and storage at −70 grade Celsius until the cytokine essay. Plasma cytokine concentrations were determined by double-ligand ELISA, as described by Bestetti et al. The protocol consisted of performing 96-well flat-bottomed microtiter plates containing the coating of 100 *μ*L/well of specific antibody (Pharmingen, Saint Louis, Mo) for TGF-beta at a concentration of 2 *μ*g/mL of coating buffer, which were incubated at 4°C overnight.

After incubation time, the plates were washed with specific kit buffer and incubated for 120 min at 37°C with a buffer containing 1% bovine serum to preclude nonspecific binding. To maintain specificities, standard curves were performed using recombinant human cytokines. Samples and standards were loaded into wells and incubated overnight at 4°C. Subsequently, the plates were washed and the appropriate biotinylated polyclonal or monoclonal anti-TGF-beta antibody (Pharmingen) was added. After 1 hour, the plates were washed 5 times with appropriate buffer followed by the addition of diluted avidin-peroxidase (Sigma). Again, the plates were then incubated for 15 min and completely washed again.

Finally, the chromogenic substrate O-phenylenediamine (OPD-Dako, Denmark) (0.4 mg OPD plus 0.4 *μ*L H_2_O_2_ for 1 mL of substrate buffer) was added. The developed color intensity was measured spectrophotometrically at 490 nm using an ELISA plate scanner (Spectra Max 250-Molecular Device, USA). The results of TGF-beta levels are expressed as pg/ml plasma.

### 2.3. Sodium Dodecyl Sulfate-Polyacrylamide Gel Electrophoresis (SDS-PAGE) Gelatin Zymography of MMP-9 and MMP-2

Gelatin Zymography was used to determine MMP-2 and MMP-9 activity in the plasma previously collected for the cytokine essay, according to Demacq et al. [25] and Fontana et al. [26] Briefly, plasma samples were diluted in sample buffer (final concentration): 2% SDS (sodium dodecyl sulfate), 125 mm Tris-HCl, pH 6.8, 10% glycerol, and 0.001% bromophenol blue, and were subjected to electrophoresis on 7% SDS-polyacrylamide gel electrophoresis copolymerized with the substrate for gelatinases, gelatin (0.05%).

After electrophoresis was completed, the gels were incubated twice for 30 min at room temperature in a 2% Triton X-100 solution, washed, and incubated at 37°C for 18 hours in Tris–HCl buffer, pH 7.4, containing 10 mmol/L CaCl_2_. Gels were stained with 0.05% Coomassie Brilliant Blue G-250 and then destained with 30% methanol and 10% acetic acid. Gelatinolytic activity was detected as an unstained band against the background of Coomassie blue-stained gelatin. Enzyme activity was assayed using Image J software by optical densitometry.

Gelatinolytic activities were normalized with regards to an internal standard (2% fetal bovine serum, FBS) to correct for loading and intergel variation, and the results were expressed as arbitrary units. The MMP-2 form was identified as band at 72 kDa and MMP-9 as band at 92 kDa. MMP-2 and MMP-9 plasma levels were studied by gel zymography, and presented as optical densities (OD).

## 3. Statistical Analysis

Data are presented as median (25% percentile, 75% percentile) because they had non-normal distribution. The Kruskal–Wallis test was used to compare continuous variables among patients with CCHD, CCHD-SAH, and controls. The Mann–Whitney test was used to compare MMP-2 and MMP-9 plasma levels in patients with CCHD versus CCHD-SAH. The Spearman correlation test was used to correlate plasma levels of MMP-2 and MMP- 9 with those of TGF-beta. In all comparisons, differences at the level of *p* < 0.05 were considered to have statistical significance.

## 4. Results

**Figure 1:**
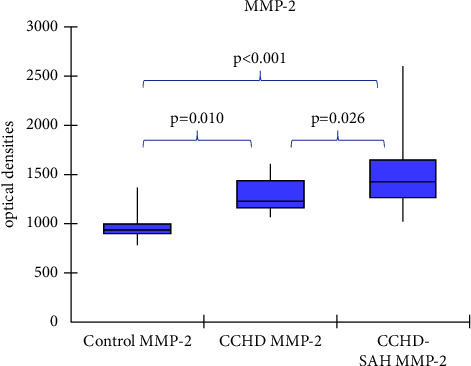
Comparison of MMP-2 plasma levels in controls, in patients with chronic Chagas heart disease (CCHD) alone, and in patients with chronic Chagas heart disease-systemic arterial hypertension (CCHD-SAH).

**Figure 2:**
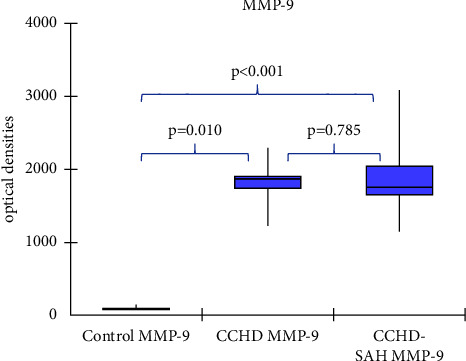
Comparison of MMP-9 plasma levels among controls, chronic chagas heart disease alone (CCHD), and chronic chagas heart disease-systemic arterial hypertension (CCHD-SAH) patients.

Median MMP-2 plasma levels were 940 OD (898.1; 1000.8) in controls, 1224.7 OD (1160; 1433.5) in CCHD patients, and 1424.1 OD (1267.5; 1561) in CCHD-SAH patients (*p* < 0.001).  Figure 1 illustrates these findings. Median MMP-2 plasma levels were increased in patients with CCHD-SAH in comparison with those with CCHD alone (*p*=0.026), as depicted in Figure 1. Median MMP-9 was 89.7 OD (80.1; 95.9) OD in controls, 1870 OD (1740; 1904.1) in CCHD patients, and 1754,6 OD (1649.5; 2049.4) in CCHD-SAH patients (*p*=0.0003), as shown in Figure 2. No difference was observed in MMP-9 plasma levels between CCHD and CCHD-SAH groups (*p*=0.785), as depicted in Figure 2. Plasma levels of MMP-9 were higher than those of MMP-2 in patients with CCHD alone (*p*=0.001) and in those with CCHD-SAH (*p*=0.01).

**Figure 3:**
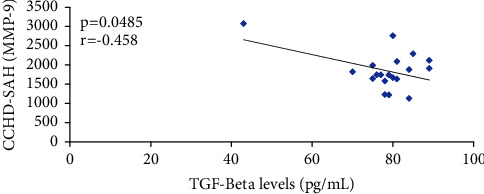
Moderate negative correlation between MMP-9 plasma levels and TGF-beta plasma levels in patients with CCHD-SAH (chronic chagas heart disease-systemic arterial hypertension).

No correlation was found between TGF-beta plasma levels and MMP2 plasma levels in patients with CCHD alone (*r* = 0.22; *p*=0.78), and in those with CCHD-SAH (*r* = 0.12; *p*=0.74). TGF-beta plasma levels did not correlate with MMP-9 plasma levels in patients with CCHD alone (*r* = 0.42; *p*=0.71); in those with CCHD-SAH, a negative moderate correlation was observed (*r* = −0.45; *p*=0.048).  Figure 3 illustrates these features. No correlation was found between MMP-2 and MMP-9 plasma levels in patients with CCHD alone, (*r* = 0.09, *p*=0.7**)** and in patients with CCHD-HAS (*r* = 0.13, *p*=0.72).

## 5. Discussion

This investigation shows that MMP-2 and MMP-9 plasma levels were higher in patients with CCHD-SAH and in those with CCHD alone in comparison with controls. MMP-2 plasma levels were higher in patients with CCHD-SAH in comparison to those with CCHD; however, MMP-9 plasma levels were similar in patients with CCHD alone and in patients with CCHD-SAH. MMP-9 plasma levels were higher than MMP-2 plasma levels in both groups. Furthermore, no correlation was found between TGF-beta plasma levels and MMP-2 plasma levels, and a moderate negative correlation was observed between MMP-9 plasma levels and TGF-beta plasma levels in patients with CCHD-SAH. These findings expand our view about the ECM remodeling process in patients with CCHD-SAH because MMPs have scarcely been previously studied in patients with this condition.

Along with tissue inhibitors, MMP-2 and MMP-9 are important players in the balance of synthesis and degradation of ECM, thus potentially leading to ventricular remodeling. Some studies concerning MMP-2 and MMP-9 plasma levels have been performed in patients with CCHD alone. As far as we know, however, only one has previously been performed in patients with CCHD-SAH.

Saldivia et al. have studied MMPs plasma levels in patients with chronic CCHD-SAH. They observed that MMP-9 plasma levels were higher than those found in controls, but the same was not observed regarding MMP-2 plasma levels [21]. Conversely, we found that both MMP-2 and MMP-9 plasma levels were higher in patients with CCHD-SAH in comparison with controls. The reasons for this discrepancy are difficult to account for. One possible explanation is that CCHD-SAH patients studied by Garcia-Saldivia et al. [21] had left ventricular hypertrophy, a condition associated with increased MMP-9, whereas a concomitant LVSD was observed in our patients in the vast majority of cases. Furthermore, in comparison with controls, the increased levels of MMP-2 in our study are similar to that found in non-Chagas disease patients with HFREF [18–20].

MMP-9 serum levels were higher than MMP-2 serum levels in patients with both CCHD alone and CCHD-SAH. This finding is consistent with the clinical severity of our patients, for they had mild CCHD-SAH (only a small decrease in the LVEF), as well as with previous studies carried out in patients with CCHD in which MMP-9 serum levels were higher than MMP-2 serum levels, and in non-Chagas disease patients with SAH-induced left ventricular hypertrophy [27, 28]. Another potential explanation for this result is a reduced MMP-2 serum levels induced by ACEI, which have been found to reduce MMP-2 levels in an experimental model of heart failure [29]. However, ACEI has also been found to reduce both MMP-2 and MMP-9 levels in another experimental model of heart failure [30]. Carvedilol has also been reduced the levels of MMP-2 and MMP-9 an animal model of acute myocardial infarction [31]. However, in an extensive review, Fontana et al. did not observe any effect of ACEI or amlodipine (which were used in the treatment of our patients) on the MMP2-serum levels. Therefore, on the basis of Fontana et al. [28] review, it appears that the reduced levels of MMP-2 and MMP-9 are not the consequence of antihypertensive medication.

The fibrotic process in patients with CCHD-SAH, similarly to what appears to occur in patients with CCHD alone [32], is complex and not fully understood. MMP-2 has been associated with replacement fibrosis in the early stage of myocardial involvement by facilitation of collagen 3 deposition in the ECM. Conversely, MMP-9 has been associated with collagen I deposition, thus leading to reactive myocardial fibrosis. Therefore, our findings that MMP-9 plasma levels in patients with CCHD-SAH were similar to those found in patients with CCHD alone, and higher than MMP-2 plasma levels, suggest that reactive (interstitial) fibrosis also play a role in the ECM remodeling process in patients with CCHD-SAH, as observed in patients with CCHD alone [33]. We concede, however, that this assumption must be confirmed in morphological studies in the future.

In our study, increased levels of MMP-9 plasma levels are consistent with myocardial fibrosis consequent to collagen interstitial accumulation. Collagen deposition in the interstitial myocardium is related to several types of cytokines that regulate the production and degradation of ECM; the most important seems to be the sustained production of TGF-beta [34]. In addition, TGF-beta may also contribute to the fibronectin synthesis, another component of ECM [35]. Nonetheless, cytokines plasma levels have rarely been studied in patients with CCHD-SAH.

Rodrigues-Angulo et al. did not find any difference between plasma levels of interferon-gamma, IL-17, TNF, IL-4, IL-6, Il-2, and IL-10 in patients with CCHD-SAH in comparison to those with CCHD alone [12]. Conversely, we clearly observed increased levels of proinflammatory and anti-inflammatory cytokine levels in patients with mild CCHD-SAH in comparison with CCHD patients alone. Of interest, TGF-beta plasma levels were increased in patients with CCHD-SAH with mild disease in comparison to patients with CCHD alone and in controls, thus reflecting a potential for myocardial interstitial fibrosis appearance [11].

Some authors claim that TGF-beta plays a pivotal role in the progression of CCHD. TGF-Beta plasma levels are 20-fold higher in infected patients in comparison with healthy controls. Furthermore, TGF-Beta plasma levels are higher in the indeterminate stage (the period before the appearance of myocardial involvement) of chronic Chagas disease in comparison with controls, and in patients with cardiac disease without left ventricular dysfunction in comparison with those in the indeterminate stage of the disease [36]. Its role in the pathogenesis of patients with CCHD-SAH, however, is unknown. For this reason, we correlated TGF-beta plasma levels with MMP-2 and MMP- plasma levels in this investigation.

We have previously shown that TGF-beta plasma levels are higher in patients with CCHD-SAH in comparison with patients with CCHD alone and healthy controls [11]. In the present study, no correlation between MMP-2 and TGF-beta was detected, but a moderate negative correlation of MMP-9 plasma levels and TGF-beta plasma levels was observed. This suggests that, in patients with CCHD-SAH, TGF-beta may not be a fundamental player in the interstitial myocardial fibrotic process.

This is in line with our previous study in which kinins plasma levels were increased in patients with CCHD-SAH in comparison with patients with CCHF and controls. Increased nitric oxide plasma levels were also observed in patients with CCHD-SAH in that study. These substances may increase protein kinase G levels, thus halting the TGF-beta to play a role on the fibrotic process [37].

Our study is also in line with a morphological study carried out in patients with CCHD alone with HFREF. In fact, fragments of myocardium from 19 patients with that condition were used to study growth factors by the immunoperoxidase technique (PDGF-A, PDGF-B, TGF-Beta 1, and GN-CSF) and TGF-beta. No correlation was found between TGF-beta and myocardial fibrosis, but only with PDGF-1 and the amount of inflammatory myocardial cells [38]. It is, therefore, conceivable that the same phenomenon may occur in patients with CCHD-SAH.

Our findings may contribute to the understanding of the pathogenesis of patients with CCHD-SAH, which is still much unknown. In patients with CCHD alone, the interplay of autoimmunity, microvascular coronary disease, and autonomic dysfunction is believed to play a pivotal role in the pathogenesis of the disease [39]. This may occur in patients with CCHD-SAH as well. Intracardiac sympathetic overactivity can be observed in patients with CCHD without HFREF [40] on the basis of the presence of myocytolysis (a marker of sympathetic-induced myocardial lesion) on morphological grounds.

TGF-beta plasma levels are increased in patients with SAH as a result of circulating angiotensin-induced ECM remodeling [41]. Betablockers and ACEI/ARB have successfully been used in patients with CCHD with HFREF [42]. Therefore, the use of ACEI/ARBS and betablockers in our patients with CCHD-SAH may account, at least in part, for the lack of significant correlation between TGF-beta levels and MMP-9 plasma levels, thus impeding aggravation of ECM remodeling.

In conclusion, patients with CCHD-SAH who were treated with antihypertensive medication have increased MMP-2 and MMP-9 plasma levels. Further research is needed in this direction.

## Figures and Tables

**Table 1:** Clinical characteristics of Chagas disease patients enrolled in the study.

	CCHD (*n* = 19)	CCHD-SAH (*n* = 14)
Age (years)	62 ± 2	63 ± 2
Male	11 (57.9%)	9 (64.2%)
Female	8 (42.1%)	5 (35.8%)
Abnormal ECG	17 (89%)	13 (93%)
Reduced LVEF	16 (86%)	13 (93%)
LVEF (%)	54 ± 1.6	53.5 ± 1.3

Age (mean ± standard deviation); Male: number (percentage); ECG: 12 lead-electrocardiogram; LVEF *=* left ventricular ejection fraction.

## Data Availability

No data were used to support this study.
